# Draft Genome Sequences of Three Flavobacterium psychrophilum Strains Isolated from Diseased Ayu (Plecoglossus altivelis altivelis) Caught at Three Sites in the Kagami River in Kochi, Japan

**DOI:** 10.1128/MRA.00773-19

**Published:** 2019-08-22

**Authors:** Hazuki Yamashita, Takayuki Wada, Yusuke Kato, Takuji Ikeda, Masayuki Imajoh

**Affiliations:** a Graduate School of Integrated Arts and Science, Kochi University, Nankoku, Kochi, Japan; b Department of International Health, Institute of Tropical Medicine, Nagasaki University, Nagasaki, Japan; c School of Tropical Medicine and Global Health, Nagasaki University, Nagasaki, Japan; d Laboratory of Fish Disease, Aquaculture Course, Department of Marine Resource Science, Faculty of Agriculture and Marine Science, Kochi University, Nankoku, Kochi, Japan; University of Arizona

## Abstract

Flavobacterium psychrophilum is a Gram-negative, psychrophilic bacterium within the family Flavobacteriaceae. Here, we report the draft genome sequences of three F. psychrophilum strains isolated from skin ulcers of diseased ayu caught by tomozuri angling at three sites in the Kagami River in Japan.

## ANNOUNCEMENT

Ayu (Plecoglossus altivelis altivelis) is a popular target for angling using the Japanese fishing method known as tomozuri. The Kagami River is located in the central Kochi Prefecture on Shikoku Island in Japan and is 31 km long. A large number of juvenile ayu reared in hatcheries are released annually into the river in order to enhance the natural stocks of ayu. The Kagami Dam was built in the middle of the river, dividing it into two streams and preventing the ayu from going upstream or downstream.

We caught over 100 ayu by tomozuri angling in May and June of 2017 at the following three sites: one in the lower reaches of the dam, one near the dam, and one in the upper reaches of the dam. There were some individuals with skin ulcers, a clinical sign of bacterial cold-water disease (1), among those caught at each site. The diseased individuals were identified as released ayu by discriminant analysis in our previous study (2). Samples were taken from the skin lesions and streaked onto modified cytophaga (MCYT) agar (3). The agar plates were incubated at 18°C for 7 days. A single colony was picked from each plate and cloned by subculturing onto MCYT agar. Here, we report the draft genome sequences of three Flavobacterium psychrophilum isolates, namely, strain KKAC-1706 from the site in the lower reaches of the dam, strain KKOK-1706 from the site near the dam, and strain KHIR-1705 from the site in the upper reaches of the dam.

Single colonies of three strains were picked into 10 ml of MCYT broth and grown for 4 days at 18°C. Following centrifugation at 10,000 × *g* for 10 min at 4°C, genomic DNA samples were prepared using Genomic-tip 500/G columns and the recommended buffer system (Qiagen) according to the manufacturer’s instructions. Library construction was performed according to the Illumina TruSeq nano DNA library prep kit protocol and was sequenced on the Illumina MiSeq platform using the MiSeq reagent kit v3 (600 cycle) with 300-bp paired-end reads. Raw reads were imported into CLC Genomics Workbench 9.5.3 (Qiagen) with default parameters for trimming and removing low-quality base calls (quality limit of 0.05 and minimum read length of 50 bp) and *de novo* assembly of high-quality reads. Assembly conducted by the software is based on de Bruijn graph algorithm. The assembled data were annotated using the DDBJ Fast Annotation and Submission Tool v. 1.1.0 (4). Table 1 shows sequencing metrics and genomic data of three strains. The Flavobacterium psychrophilum multilocus sequence typing (MLST) databases (http://pubmlst.org/fpsychrophilum/) were used for MLST analysis of the three strains. From this analysis, strains KKOK-1706 and KHIR-1705 were classified into MLST sequence type 52 (ST52), whereas strain KKAC-1706 was classified into ST45. The two STs belong to two lineages known to infect ayu in Japan (5).

**TABLE 1:** Sequencing metrics and genomic features of three F. psychrophilum strains

Strain	No. of raw reads	Avg length (bp) of raw reads	No. of high-quality reads	Avg length (bp) of high-quality reads	Coverage (×)	G+C content (%)	No. of contigs	Genome size (bp)	No. of coding sequences	No. of tRNAs	No. of rRNAs	GenBank accession no.	SRA accession no.
KKAC-1706	1,817,338	300.6	1,656,941	236.2	166	32.5	160	2,680,547	2,331	38	3	BJTD00000000	DRR182487
KKOK-1706	2,331,724	300.6	2,188,898	243.9	247	32.4	153	2,730,491	2,387	39	3	BJTE00000000	DRR182488
KHIR-1705	2,113,688	300.6	1,978,561	241.3	226	32.4	148	2,730,165	2,389	39	2	BJTF00000000	DRR182489

### Data availability.

This whole-genome shotgun project and raw sequencing reads have been deposited in DDBJ/GenBank and the DDBJ/Sequence Read Archive, respectively. The respective accession numbers are listed in Table 1.
